# Long-term results of robotic radiosurgery for non brachytherapy patients with cervical cancer

**DOI:** 10.1007/s00066-020-01685-x

**Published:** 2020-09-24

**Authors:** Janis Morgenthaler, Christhardt Köhler, Volker Budach, Jalid Sehouli, Carmen Stromberger, Angela Besserer, Maike Trommer, Christian Baues, Simone Marnitz

**Affiliations:** 1 Faculty of Medicine and University Hospital Cologne, Department of Radiation Oncology and Cyberknife Center, University of Cologne, Kerpener Str. 62, Cologne, 50937 Germany; 2 Center for Integrated Oncology (CIO Köln Bonn), Kerpener Str. 62, Cologne, 50937 Germany; 3 Department of Gynecological Oncology, Asklepios Clinic Hamburg Altona, Paul-Ehrlich-Straße 1, Hamburg, 22763 Germany; 4 Department for Radiation Oncology, Campus Virchow-Clinic, Charité—University Medicine Berlin, Augustenburger Platz 1, Berlin, 13353 Germany; 5 Department of Gynecology, Campus Virchow-Clinic, Charité—University Medicine Berlin, Augustenburger Platz 1, Berlin, 13353 Berlin Germany

**Keywords:** Cyberknife, Stereotactic Radiotherapy, Boost, Stereotactic Body Radiotherapy, SBRT

## Abstract

**Background:**

Consolidation brachytherapy is a critical treatment component for cervical cancer patients undergoing primary chemoradiation. Some patients are unsuitable for brachytherapy for a variety of reasons. The use of alternatives (LINAC-based stereotactic radiosurgery or external beam boosts) compromise oncologic results in cervical cancer patients. Thus, we evaluated the value of brachytherapy-like doses prescriptions using robotic radiosurgery (CyberKnife®, CR, Acuuray, Sunnyvale, CA, USA).

**Methods:**

From 06/2011 to 06/2015, 31 patients (median age 53 years; range 30–77 years) with histologically proven FIGO stages IB-IVB cervical cancer underwent primary chemoradiation. All patients were either not suitable for intracervical brachytherapy for a variety of reasons or refused the brachytherapy. To achieve an adequate dose within the tumor, a CK boost was applied after fiducial implantation. In 29 patients, a dose of either five times 6 Gy or five times 5 Gy was prescribed to the target volume. Two patients received three times 5 Gy. The target dose was prescribed to the 70% isodose. Treatment toxicity was documented once weekly regarding vaginal mucositis, bladder, and bowel irritation according to CTCAE v. 4.03. If possible 3 months after completion of treatment intracervical curettage was performed to exclude residual tumor and the patients were followed up clinically. Sparing of organs at risk (OAR) and outcome in terms of local control (LC), overall survival (OS), and progression-free survival (PFS) were assessed.

**Results:**

Of the 31 patients, 30 have completed CK boost therapy. The median follow-up time was 40 months (range 5–84 months). General treatment tolerability was good. Except for 1 patient, who had diarrhea grade 3, no treatment related side effects above grade 2 were reported. Sparing of OAR was excellent. The 1‑, 3‑, and 5‑year OS rates were 89, 60, and 57% respectively across all stages. Seven patients showed progression (28%), only two of them with local relapse (8%), resulting in an LC rate of 92% after 3 and 5 years. Mean PFS was 41 months (range 2–84 months). Patients with local recurrence had PFS of 5 and 8 months. Five patients developed distant metastases. Fifteen patients (48%) underwent intracervical curettage 3 months after completion of treatment of which 14 (93%) had complete pathologic response.

**Conclusion:**

Brachytherapy remains the standard of care for patients diagnosed with cervical cancer and indication for primary chemoradiation. In terms of local control, CyberKnife®-based boost concepts provide excellent local control. It can be an alternative for patients who cannot receive adequate brachytherapy. Distant relapse still remains a challenge in this context.

## Introduction

Intracervical brachytherapy remains an integral part of the definitive chemoradiation in cervical cancer patients. It provides the possibility to cover the macroscopic tumor with biologically equivalent doses with EQD2 ≥85 Gy. There is a clear EQD2 response relationship with regard to oncologic results and toxicity [1, 2]. For several reasons, there has been a decreased use of brachytherapy for years. While in the USA at the beginning of the new millennium, changes in reimbursement led to a dramatic decline of brachytherapy use, different factors like decentralization, privatization, and ambulantization of treatment facilities may play a role that intracervical brachytherapy as a very personnel intensive and resource binding treatment continues to disappear more and more [3–5].

LINAC-based stereotactic radiosurgery is not considered as an appropriate alternative for intracervical brachytherapy [6] because prescribed doses mostly remain below the recommended EQD2 of at least 85 Gy. Benchmark parameters have been defined including EQD2 ≥85 Gy, the concomitant use of cisplatin, the implementation of brachytherapy for curative chemoradiation, treatment in specialized centers of at least 28 patients with primary chemoradiation, and an appropriate treatment time of not more than 8 weeks overall treatment time [2, 7–12].

The renunciation of brachytherapy led to dramatically worse survival curves in cervical cancer patients [5]. Nevertheless, there are some clinical situations in which brachytherapy cannot be performed: Patient refusal of brachytherapy, recurrent dislocation of the Smit sleeve or inability of insertion, the presence of poorly responsive or asymmetric tumors or uterus bi-cornis and/or bi-collis, in which a satisfying target coverage cannot be achieved with the brachytherapy equipment available at the institution (2-channel-based tandem brachytherapy applicator). For those patients, we evaluated the role of robotic radiosurgery (CyberKnife®) boost concepts. The nonisocentric technique of the CyberKnife® allows even complex target volumes with steep dose gradients to be irradiated excellently. The aim of the single institution study was to evaluate the feasibility, local control, and survival of patients with a brachytherapy-like dose prescription.

Published data on stereotactic radiation addressed the use as an alternative for brachytherapy or recurrent disease or oligometastatic disease [13–21]. To our knowledge, no outcome data are available in which stereotactic boost techniques as alternative to brachytherapy have been systematically analyzed in the primary situation.

## Methods and patients

From 2011–2015, 31 patients were included after a positive votum of the Ethical Committee and the Agency for Radiation Protection. All patients were aware of the experimental character of the treatment and gave their informed consent. Patient characteristics are summarized in Table 1. Indications for CyberKnife® boost treatment were uterus bi-collis and bi-cornis (*n* = 1), refusal of Smit sleeve insertion (*n* = 3), refusal of brachytherapy (*n* = 7) and inability to find the cervical os and implant the Smit sleeve or loss of Smit sleeve and refusal of reinsertion (*n* = 22).

**Table 1:** Patient characteristics

Patient No.	Age at diagnosis (years)	Follow-up (months)	FIGO	Pelvic nodes	Para-aortic nodes	Site of first recurrence
*1*	32	51	IB	Yes, pN1 (1/32)	No	No progression
*2*	35	66	IB	Yes, pN1 (4/32)	No	No progression
*3*	30	44	IIA	Yes, pN1 (4/25)	No	Multiple
*4*	71	12	IIA	Yes, radiologic	No	Local
*5*	55	84	IIB	No, radiologic	No	No progression
*6*	61	79	IIB	No, radiologic	No	No progression
*7*	47	32	IIB	No, pN0 (0/39)	No	Unknown
*8*	47	63	IIB	No, radiologic	No	No progression
*9*	33	8	IIB	Yes, pN1 (9/20; ece+)	No	Unknown
*10*	62	49	IIB	No, radiologic	No	No progression
*11*	46	73	IIB	Yes, pN1 (1/19)	No	No progression
*12*	63	30	IIB	No, radiologic	No	Unknown
*13*	46	8	IIB	Yes, pN1 (17/23)	No	Unknown
*14*	73	49	IIB	No, radiologic	No	Unknown
*15*	48	39	IIB	Yes, pN1 (4/32)	No	No progression
*16*	39	45	IIB	No, radiologic	No	No progression
*17*	32	50	IIB	No, radiologic	No	No progression
*18*	44	36	IIB	No, radiologic	No	No progression
*19*	53	13	IIB	No, pN0 (0/32)	No	No progression
*20*	57	21	IIB	No, radiologic	No	Local
*21*	65	49	IIB	No, pN0 (0/31)	No	No progression
*22*	58	64	IIB	Yes, radiologic	No	No progression
*23*	77	11	IIIB	No, radiologic	No	Multiple
*24*	67	66	IIIB	No, radiologic	No	No progression
*25*	74	35	IIIB	No, radiologic	No	Multiple
*26*	65	12	IIIB	No, radiologic	No	Liver
*27*	32	58	IVA	Yes, pN1	Yes, pM1 (LYM)	No progression
*28*	51	76	IVA	Yes, pN1 (9/40)	Yes, pM1 (LYM)	No progression
*29*	57	30	IVA	Yes, radiologic	Yes	Lung
*30*	57	14	IVA	Yes, pN1 (24/35)	Yes, pM1 (20/21) (LYM)	Unknown
*31*	49	12	IVA	Yes, radiologic	No	Lymph nodes
*Mean*	*52 (30–77)*	*41 (8–84)*	*–*	*–*	*–*	*–*

*LYM* lymph node metastasis, *ECE+* extracapsular extention

The oncologic outcome was analyzed using the endpoints overall survival (OS), progression-free survival (PFS), and local control (LC).

The institutional standard consists of chemoradiation with intracervical brachytherapy. The patients usually receive a Smit sleeve implantation in the third or fourth week of treatment. We perform another MRI (magnetic resonance imaging) with the sleeve in place. Brachytherapy equipment consists of a 2-channel pin/ring applicator (Varian). For brachytherapy, patients are placed on a gynecological chair with an empty rectum, the applicator is inserted and the vagina is stuffed with gauze bandages and a rectal probe is inserted. A CT (computed tomography) scan then is performed and fused with the current and pretherapeutic MRI. The planning target volume (PTV) consists of the cervix and the residual tumor from the T2-weighted MRI sequence. The image information from the pretherapeutic MRI is always cointerpreted and it is up to the treating radio-oncologist to adjust the target volumes accordingly.

### Treatment planning and delivery

CT-planning for external beam radiation (EBRT) was done in supine position with emptied rectum and filled bladder with kneefix and footfix on a big bore TOSHIBA CT. Primary EBRT chemoradiation was performed. It included) with 6/15 MV photons using volumetric arc techniques (VMAT) on a linear accelerator (DHX, Varian) or TomoTherapy (Accuray Inc.). Five weekly single doses of 1.8 Gy to the primary tumor including the uterus, pelvic, and in case of histologically confirmed para-aortic lymph nodes including the para-aortic node up to the renal vessels to a total dose of 50.4 Gy in 28 fractions were given. A simultaneous boost was given with five weekly single doses of 2.12 Gy to both parametric regions to a total dose of 59.36 Gy in 28 fractions to all patients. Cisplatin 40 mg/m^2^ body surface area was administered once weekly for five applications.

In the 3rd or 4th week of EBRT, a second gynecologic examination was performed and three to four fiducials were implanted to the right and left anterior and posterior tumor border.

Two to three days after fiducial implantation, CT and MRI were performed in the treatment position. Patients emptied rectum prior to scanning. All patients were placed in the supine position on a 2-inch foam mat to enhance patient comfort. A knee rest and foot rest were added to stabilize the pelvic region. CT images were acquired with 1‑mm slice thickness including the pelvic region as well as the lumbar vertebra 4 (L4) and lumbar vertebra 5 (L5) vertebrae, which is necessary for later digitally reconstructed radiograph (DRR) generation and spinal alignment. The MRI of the pelvis was conducted (T1 + gadolinium contrast, T2) with the patient in the same position as in the CT scan. Contouring for the boost techniques was performed on CT and MRI images, which were fused using bony structures and fiducial clips. The PTV was defined as the cervix including the residual tumor as identified on T2-weighted MRI, the parametric region, and/or the corpus uteri. In analogy to brachytherapy (BT), no margin was added.

The CyberKnife® boost was performed in the 4th to 6th week of treatment. An “Xsight Spine Setup Plan” was created to optimize the initial treatment alignment, defining the lower part of the lumbar spine as the tracking target. With this setup plan, the patient can be positioned according to the bony structures of the spine. By moving the treatment couch, the patient is aligned so that the translational and rotational positional deviations are corrected. This step facilitates the finding of the cervical target. After spinal alignment, the current treatment plan was loaded and the patient was brought into the actual treatment position. With the help of translatory table movements, which were previously calculated from the location of the treatment center in the planning software, the patient was brought into the treatment position without rotation changes occurring. The dose was prescribed to the 70% isodose to allow higher doses within the target volume like conventional brachytherapy. Small subvolumes with up to 200% of the prescribed dose were allowed.

Five fractions to a total dose of 25–30 Gy were prescribed comparable to the institutional brachytherapy concept. The institution changed the brachytherapy concept between 2011 and 2015 from 5 × 6 Gy to 5 × 5 Gy, which is why some patients received 30 Gy and others 25 Gy boost dose. Two or three weekly robotic radiosurgery fractions were given (every other day) overlapping with external beam radiation. This procedure has already been described in earlier work as safe and reliable and with favorable dose–volume histogram (DVH) parameters [16, 17].

Treatment planning was carried out using Multiplan® 4.5 (Accuray Inc.) planning workstations. Inverse planning was performed to obtain the optimal dose distribution.

The rectal wall, bladder wall, and sigmoid wall were contoured as the outer wall of the organs minus 2 mm for the inner wall so that a ring structure was created. The small intestine was defined as the entire abdominal cavity without other organs at risk (OARs), musculature or the planning target volume (PTV) up to the fourth lumbar vertebra. Biologically effective doses of EBRT and CyberKnife were calculated with α/β = 3 for normal tissue.

For the CyberKnife boost, organ walls were generated from rectum, sigma and bladder like for EBRT. The EQD2 (α/β = 3) on 0.1, 2 and 5 ccm of rectal wall, bladder wall and sigma wall were calculated from DVHs of EBRT + CyberKnife according to Georg et al. [1]. For the EBRT (IMRT, Helical Tomotherapy or Rapid Arc) the following dose constraints were used for the whole organ: small intestine: V45 <20%, V20 <40%, Dmean <30 Gy; rectum: V40 <70%, V50 <50%; bladder: V30 <60%, V50 <30%; femoral heads: Dmean <40 Gy.

For the organs at risk, EQD2 was calculated to 0.1, 2, 5 cc of the rectal, sigmoid and bladder wall, respectively for robotic radiosurgery and EBRT (Tables 2 and 3). For the target volume, V100, V90, D100, D90, D_max_, D_mean_, Conformity Number (CN) and Conformity Gradient Index (CGI) were analyzed (Table 4).

**Table 2:** EQD2 for CyberKnife® deposed to subvolumes of the critical organs at risk (OAR) (rectum, sigmoid, bladder)

Subvolume/Organ	Dose to subvolume (Gy) EQD2 (Gy) to OARs (CyberKnife)
*Rectal wall*	
D 0.1cc	26.9 ± 4
D 2.0cc	13.8 ± 2.6
D 5.0cc	6.2 ± 2.2
*Sigmoid wall*	
D 0.1cc	22.8 ± 5
D 2.0cc	11.4 ± 3.6
D 5.0cc	6.2 ± 1.9
*Bladder wall*	
D 0.1cc	36.2 ± 5.5
D 2.0cc	25.7 ± 4.3
D 5.0cc	15.4 ± 2.4

**Table 3:** Organ doses for external beam radiation (EBRT)

Organ	Dose		
	D_mean_ (Gy)	D_max_ (Gy)	D_min_ (Gy)
Small bowel	30.29 ± 4.50	57.20 ± 4.28	4.82 ± 2.22
Rectum	46.29 ± 3.91	57.81 ± 4.12	17.70 ± 10.40
Sigma	48.15 ± 4.60	56.29 ± 4.36	38.25 ± 8.74
Bladder	47.77 ± 4.65	59.28 ± 3.94	31.52 ± 8.77

*D*_*mean*_ mean dose, *D*_*max*_ maximum dose, *D*_*min*_ minimum dose

**Table 4:** Planning target volume (PTV) parameters obtained from the dose–volume histogram (DVH) analysis

Tumor parameters for CyberKnife (nominal dose in Gy ± SD; subvolumes in %)	
V_100_ (%)	96.97 ± 2.05
V_90_ (%)	99.49 ± 0.73
D_100_ (Gy)	20.75 ± 4.16
D_90_ (Gy)	28.75 ± 4.51
D_max_ (Gy)	39.69 ± 7.71
D_mean_ (Gy)	32.53 ± 5.41
CN	0.79 ± 0.07
CGI	36.88 ± 16.0

*V*_*100*_ *(%)* Volume covered by 100% of the prescribed dose, *V*_*90*_ volume covered by 90% of the prescribed dose (%), *D*_*100*_ Dose to 100% of the target volume (Gy), *D*_*90*_ Dose to 90% of the target volume (Gy), *D*_*mean*_ mean dose, *D*_*max*_ maximum dose, *D*_*min*_ minimum dose, *CN* Conformity Number, *CGI* Conformity Gradient Index

### Clinical follow-up and statistical analysis

Acute treatment-related toxicity was documented once weekly according to CTCAEv.4.03 Three months after treatment we offered intracervical curettage to the patients to exclude residual tumor. Fifteen patients (48%) underwent the procedure and all were followed up clinically. The follow-up included a gynecological examination to exclude local relapse.

Statistical analysis was performed using IBM SPSS Statistics® Version 21 software. Case numbers are too low to obtain meaningful *p*-values.

## Results

### Target volume parameters

**Fig. 1:**
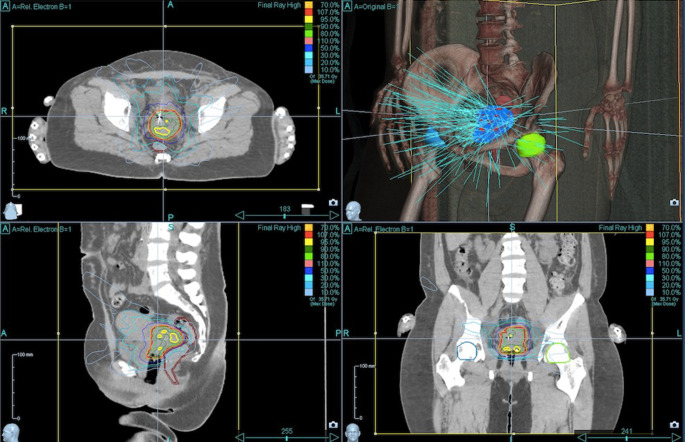
Beam geometry for CyberKnife boost for a patient with FIGO IIB. Dose distribution in axial/sagittal/frontal view

**Fig. 2:**
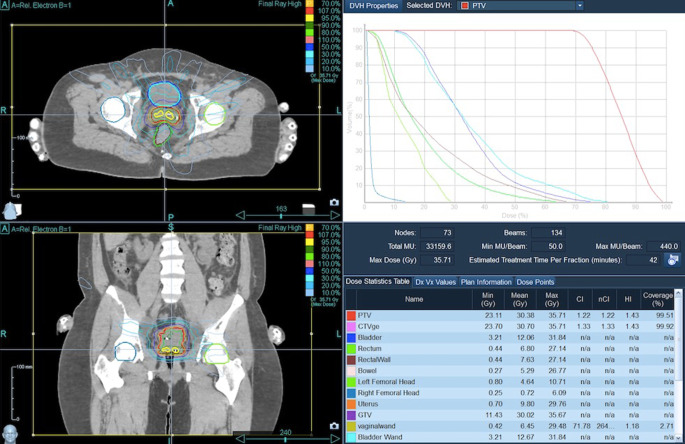
Dose distribution for the same patient as Fig. 1 including dose–volume histogram in the *upper right panel*

**Fig. 3:**
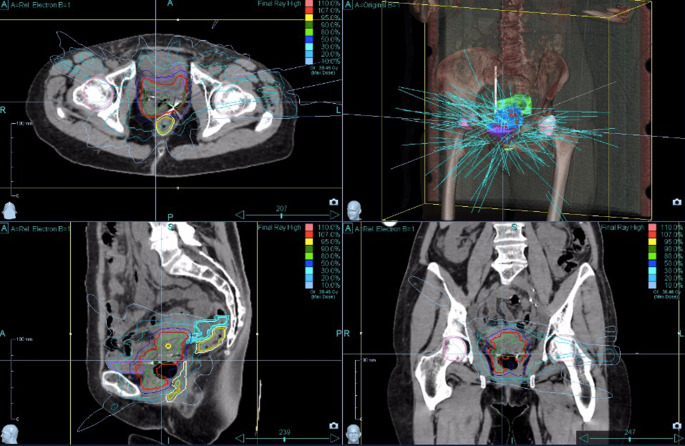
Beam geometry for CyberKnife boost for a patient with FIGO IVA. Dose distribution in axial/sagittal/frontal view

**Fig. 4:**
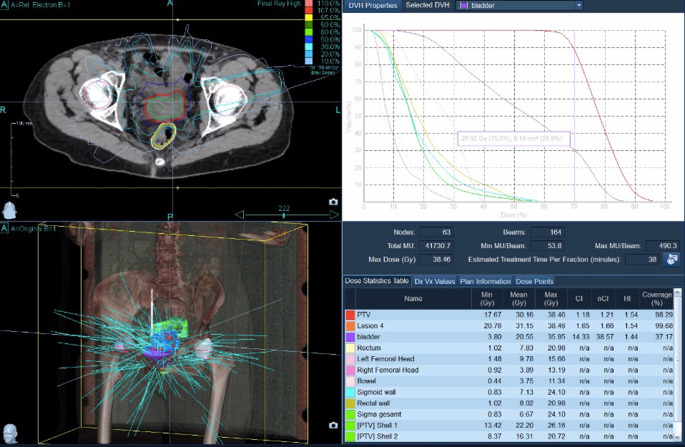
Dose distribution for the same patient as Fig. 3 including dose–volume histogram in the *upper right panel*

Target volume coverage could always be achieved. With a median V_100_ of 97% (i.e., 100% of the PTV received 97% of the prescribed dose), the coverage was almost optimal. The achieved values were comparable with the recommendations of the GEC-ESTRO Guidelines and the EMBRACE study [22] on brachytherapy planning. Figures 1–4 show dose distribution and beam geometry of the Cyberknife boost plan for two patients.

### Organs at risk

As can be seen in Tables 2 and 3, the CyberKnife® technology shows excellent sparing of the organs at risk (OAR). The dose limits, which are associated with a low risk for early and late side effects by Georg et al. and were also agreed as planning goals in the GEC-ESTRO Guidelines and the EMBRACE study [22], could be achieved.

The sparing of the OAR resulted in favorable acute and late side effects (Table 5). In the case of acute side effects, one patient showed grade 3 diarrhea in the 4th week of therapy. This was before the CyberKnife® boost therapy and was therefore not related to the technique used. This patient was hospitalized supportively and treated symptomatically. The prescribed therapy could be completed. Another patient with FIGO IV and bilateral urinary stasis suffered from sepsis in the 12th month after treatment, which was due to an abdominal focus. This patient had to have a part of her intestine resected. A connection with the CyberKnife® therapy was not seen. With regard to hematotoxicity, the usual blood count changes were observed after chemotherapy. One patient suffering from leukocytopenia and thrombocytopenia grade IV had AML in her medical history.

**Table 5:** Toxicities by CTCAE v4.05

Toxicity	Early (beginning of treatment up to 3 months after completion) (%)	Late (3 months and later) (%)
**GI**		
*None*	*N* = 5 (16)	*N* = 20 (64)
*Mild (Grade I–II)*	*N* = 21 (68)	*N* = 2 (7)
*Severe (Grade III–IV)*	*N* = 1 (3)	*N* = 1 (3)
*Not reported*	*N* = 4 (13)	*N* = 8 (26)
**GU**		
*None*	*N* = 11 (35)	*N* = 21 (68)
*Mild (Grade I–II)*	*N* = 16 (52)	*N* = 5 (16)
*Severe (Grade ≥III)*	*N* = 0 (0)	*N* = 0 (0)
*Not reported*	*N* = 4 (13)	*N* = 5 (16)
**Vaginitis**		
*None*	*N* = 9 (29)	*N* = 20 (64)
*Mild (Grade I–II)*	*N* = 20 (64)	*N* = 2 (7)
*Severe (Grade ≥III)*	*N* = 0 (0)	*N* = 0 (0)
*Not reported*	*N* = 2 (7)	*N* = 9 (29)
**Hematotoxicity**		
*Leukopenia*		
None	*N* = 0	–
Grade I–II	*N* = 22 (71)	–
Grade III	*N* = 8 (26)	–
Grade IV	*N* = 1 (3)	–
*Anemia*		
None	*N* = 8 (26)	–
Grade I–II	*N* = 20 (64)	–
Grade III	*N* = 3 (10)	–
*Thrombopenia*		
None	*N* = 20 (64)	–
Grade I–II	*N* = 8 (26)	–
Grade III	*N* = 2 (7)	–
Grade IV	*N* = 1 (3)	–

*GI* gastrointestinal toxicity, *GU* genitourinary toxicity, dysuria

### Oncologic outcome

A total of 28 patients (90.3%) received either 5 × 5 Gy or 5 × 6 Gy. One patient in stage FIGO IB had a strong desire to have children. Therefore a reduced dose with 3 × 5 Gy was prescribed to provide endometrium sparing. Another patient had a conisation with a small residual tumor, which was also treated 3 × 5 Gy with the CyberKnife®. The third patient developed metastases under therapy, so that the local treatment was stopped after 3 CyberKnife® therapies.

### Overall survival

**Fig. 5:**
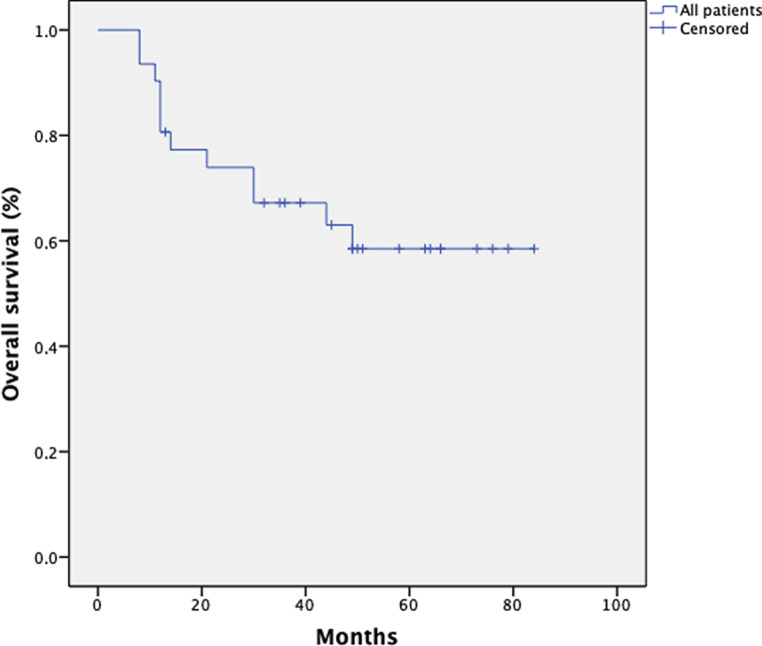
Kaplan–Meier estimates for overall survival (OS) in 31 patients

**Fig. 6:**
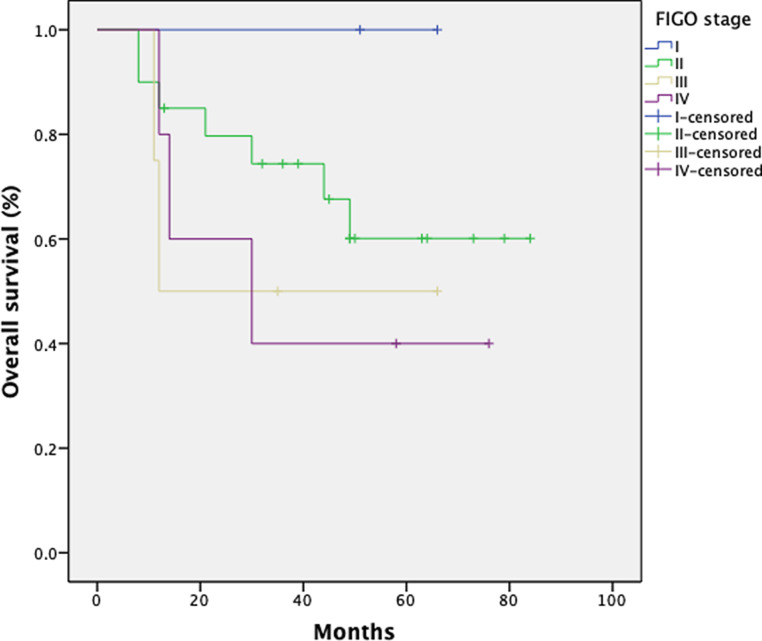
Kaplan–Meier estimates with regard to FIGO stage for 31 patients

The 1‑, 3‑, and 5‑year overall survival (OS) rates for 31 patients were 89, 60, and 57%, respectively, across all stages. Fig. 5 shows the Kaplan–Meier curve for the patient population. Fig. 6 shows OS depending on the FIGO stage. As expected, patients with a low FIGO stage had a better outcome. Two patients with FIGO stage II progressed developing distant metastasis resulting in an inferior oncologic outcome after 5 years for this subcohort (FIGO stage II: 1 year OS 88%; 3 year OS 70%; 5 year OS 50%). There is a trend for better OS in patients with negative lymph nodes.

**Fig. 7:**
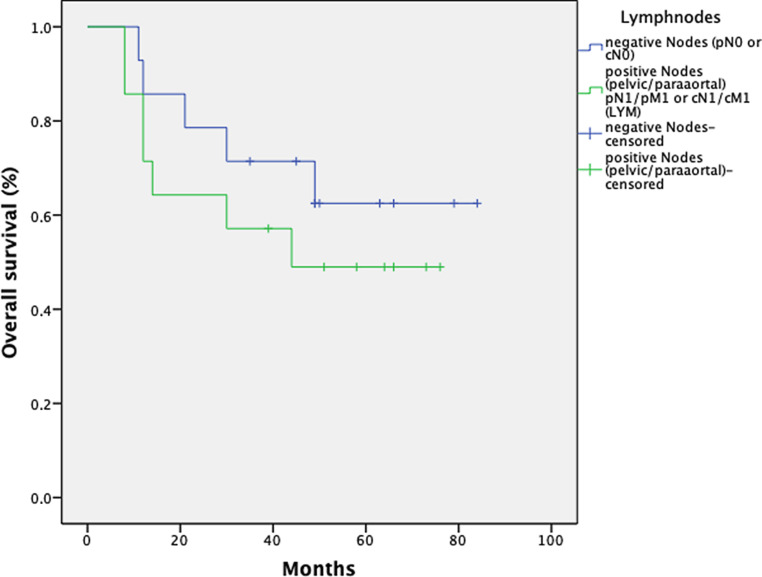
Kaplan–Meier estimates on overall survival for 28 patients with regard to the nodal status

Fig. 7 shows OS depending on the pathological or radiological lymph node status.

### Progression-free survival

**Fig. 8:**
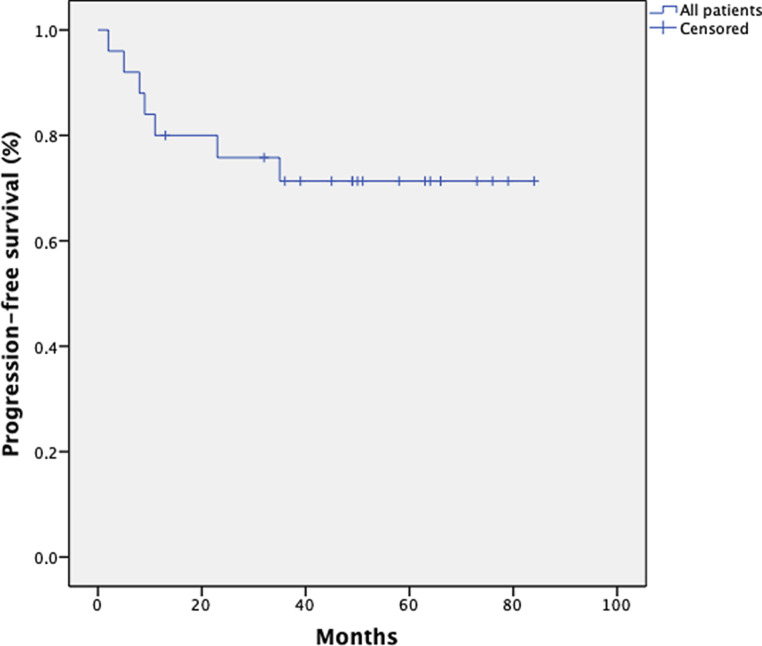
Kaplan–Meier estimates on progression-free survival (PFS) for 25 patients

**Fig. 9:**
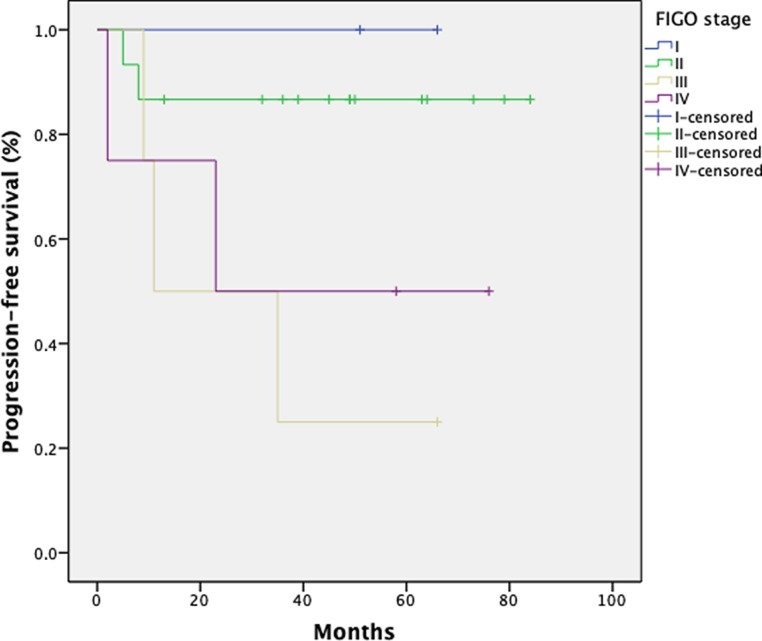
Kaplan–Meier estimates on progression-free survival (PFS) for 25 patients with regard to FIGO stage

With regard to progression-free survival (PFS), 25 patients were included in the analysis. For the missing 6 patients, sufficient data could not be collected. The mean PFS in this group was 43 months (range 2–82 months). The 1‑, 3‑, and 5‑year PFS rates were 80, 76, and 76% for all patients, respectively. Six patients (24%) developed distant metastases (*n* = 1 liver, *n* = 1 lung, *n* = 1 lymph node, *n* = 3 multiple metastases). Figs. 8 and 9 show the Kaplan–Meier curve with regard to FIGO stage.

### Local control

**Fig. 10:**
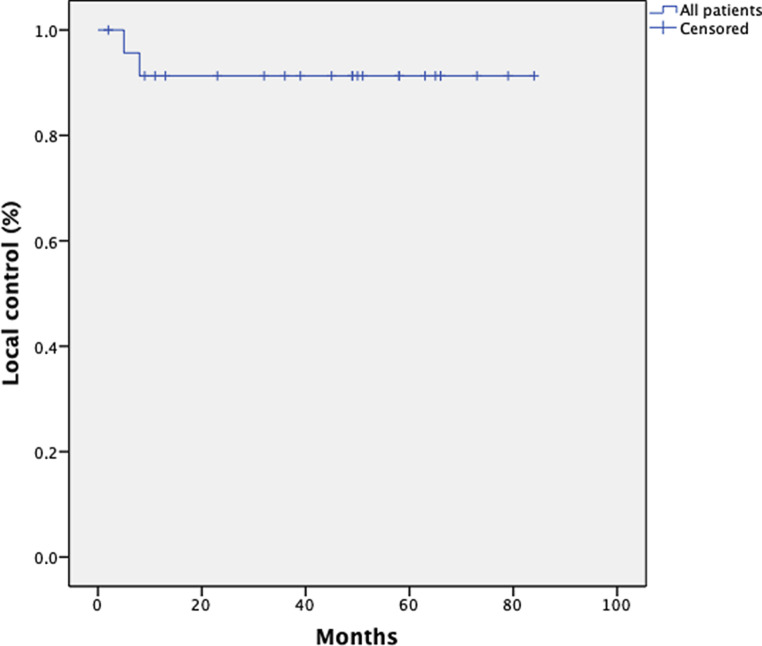
Kaplan–Meier estimates on local control (LC) for 24 patients

With regard to local control (LC), sufficient information was available for 24 patients. LC was assessed by a gynecologic examination. Only 2 patients (both FIGO stage II) showed local recurrence, resulting in an LC rate of 92% after 3 and 5 years. The patients with local recurrence had a PFS of 5 and 8 months. Fifteen (48%) of the patients received an intracervical curettage 3 months after the therapy. Fourteen (93%) patients showed pathological complete remission. Fig. 10 shows the Kaplan–Meier curve for local control.

## Discussion

For cervical cancer treatment, intracervical brachytherapy with or without interstitial brachytherapy provides the delivery of highly effective doses to the target volume. It is and remains the standard approach for treating cervical cancer patients with the indication for primary chemoradiation. The use of 3D planned MRI-guided brachytherapy led to excellent local control rate in sophisticated centers and experienced hands [23]. In the real world, there has been declining use of brachytherapy [5]. In Germany it is difficult to find adequate brachytherapy with high-tech BT equipment that meet the requirements of the GEC-ESTRO recommendations. Most patients in countries with a non-centralized oncologic care system are treated in low volume facilities [24, 25]. Against this background, a proof of principle evaluation was done with a plan comparison of 3D brachytherapy, a moderately and highly inhomogeneous dose distribution with robotic radiosurgery [17, 26]. The most visible difference was a significantly superior target coverage and less dose distribution to the critical organs at risk in favor for robotic radiosurgery, as well as the mean and maximum doses, which were lower in the robotic radiosurgery plans. It is an open question whether a maximum dose of more than 800 Gy as shown for brachytherapy really contributes to better local control or whether the avoidance of cold spots with an adequate target coverage is of greater importance. Tomé et al. calculated that dose deficit to a 1% volume of the target that is larger than 20% of the prescription dose may lead to serious loss of tissue control probability, particular attention has to be paid to small-volume cold regions in the target, even if new technologies provide the chance for better coverage [27, 28]. The present study is the first which provides long-term clinical outcome data for robotic radiosurgery as an alternative for brachytherapy boost. We could demonstrate that in all but one patient after curettage a complete histologic response could be achieved.

Several other authors addressed the use of robotic radiosurgery in cervical cancer patients. Haas et al. reported retrospectively on 6 consecutive patients (mean age 80 years) with anatomic or medical conditions precluding a tandem and ovoid boost were treated with combined external beam radiation and CyberKnife® boost to the cervix [14]. CyberKnife® was delivered with five fractions of 4 Gy single dose, one patient with 3 × 6.5 Gy. All patients tolerated the treatment well with no grade 3 or higher urinary or rectal toxicities. Grade 1/2 urinary and bowel toxicities occurred in 4 patients following conventional external beam radiation. In addition, for the 5 patients with a minimum 12-month follow-up all (100%) remain locally and distantly controlled with no evidence of disease. Otahal et al. pointed out the aspect of OAR sparing with robotic radiosurgery compared with brachytherapy. No clinical outcome data were provided in this plan comparison trial [17]. Cengiz et al. [18] focused on better target coverage with stereotactic radiosurgery with a median 99.1% target coverage by the 100% isodose line compared with 50.7% target coverage by the same isodose line in HDR brachytherapy plans (*p* < 0.05). In this study we have to take into consideration that the BT technique used is not comparable to modern image-guided BT. They only used a tandem consisting of a central applicator and two ovoids, and prescribed to point A, hence a different and outdated approach.

In the present study we could demonstrate that—in contrast to LINAC-based boost concepts—the use of a CyberKnife® system represents an alternative to intracervical ring/pin brachytherapy for unsuitable patients. The therapy is safe and feasible. The technique used allows excellent sparing of OAR with optimum target volume coverage. The side effect profile is favorable. Even after prolonged follow-up, no increased toxicity could be demonstrated in comparison to standard therapy.

With regard to local control, similar figures could be achieved as with RetroEMBRACE (91/89% after 3/5 years). Of course this comparison is highly debatable due to the low number of patients, as well as the fact that 7 patients were lost to follow-up, having 23% of the patients excluded from analysis. In a worst case scenario, all these patients developed a local relapse, resulting in an LC rate of 71% (9 of 31) instead of the 92%. With a pathological complete remission rate of over 90% in the patients who underwent curettage, this can hardly be improved. We have to take into consideration that only 15 patients (45%) were followed up pathologically.

The overall survival of 60% and 57% after 3 and 5 years is below the figures published by Quinn et al. in the 2006 FIGO report (67.6% and 59.3%; *n* = 1657) [29]. The RetroEMBRACE data also show a better overall survival after 3 and 5 years (74% and 65%; *n* = 731) [30]. However, the small number of patients in this study certainly plays a role here. Two patients with FIGO II already showed a systemic progress after a short time and died, which already had an effect on the OS of about 10% with the small number of patients. For a clean comparison on overall survival, more patients are needed.

Of course, these data have to be proven in larger phase III trials with a head-to-head comparison to standard therapy. The monocentric, single arm nature of this study make interpretation difficult. This approach is surely only suitable for very few patients. If no high tech BT equipment is available, the patients should be sent to an experienced center for adequate BT treatment. If they refuse, CyberKnife® boost represents an alternative superior to LINAC-based boost concepts.

## Conclusion

This is the first study presenting outcome data from 31 patients with cervical cancer treated with robotic radiosurgery as brachytherapy alternative. Robotic radiosurgery (RSS) provided excellent target coverage while fulfilling constraints of the critical organs at risks. RRS can be an alternative for patients who cannot undergo intracervical ring/pin brachytherapy.
